# Supplementary material for: The role of sex work laws and stigmas in increasing HIV risks among sex workers
Source: Nat Commun. 2020 Feb 18;11:773. doi: 10.1038/s41467-020-14593-6 (PMC7028952; doi:10.1038/s41467-020-14593-6)
Supplement: Supplementary file 1 — Supplementary Information [file 41467_2020_14593_MOESM1_ESM.pdf]

Supplementary Information

Title: The Role of Sex Work Laws and Stigmas in Increasing HIV Risks Among Sex Workers

Lyons et al.

Supplementary Table 1: HIV infection and country level legal status with random sample of Cameroon

	Living with HIV								
	n/N	%	X^2 p value	OR	P value	95% CI	aOR*	P value	95% CI
Legal status of sex work			<0.001						
Partially legalized	219/1895	11.6		Ref	Ref	Ref	Ref	Ref	Ref
Selling not specified	248/1266	19.6		1.86	0.103	0.88,3.94	2.31	0.029	1.09,4.90
Criminalized	1225/2521	48.6		7.23	0.001	2.19,24.22	9.69	0.001	2.60,36.11

*Adjusted for age, education level, marital status, years in sex work, clustered by country and site

Supplementary Table 2: Stigma and knowledge of living with HIV

		Knowledge of living with HIV				
		Not		Yes		X^2 p value
Stigma		n/N	column %	n/N	column %	
Perceived	Family exclusion	542/4758	11.4	235/1194	19.7	<0.001
Perceived	Family gossip	917/4778	19.2	327/1198	27.3	<0.001
Perceived	Friend rejection	563/4731	11.9	258/1198	21.5	<0.001
Anticipated	Afraid of seeking health services	552/4807	11.5	200/1207	16.6	<0.001
Anticipated	Avoided seeking health services	400/4405	9.1	76/1029	7.4	0.083
Perceived	Mistreated in health center	110/4775	2.3	60/1207	5.0	<0.001
Enacted	Health care provider gossip	200/4776	4.2	82/1206	6.8	<0.001
Enacted	Denied health services	49/4809	1.0	28/1207	2.3	<0.001
Perceived	Police refused protection	611/4718	13.0	318/1202	26.5	<0.001
Perceived	Scared in public places	620/4511	13.7	161/963	16.7	0.017
Enacted	Verbally harassed	2206/4408	50.1	572/1030	55.5	0.002
Enacted	Blackmailed	1570/4808	32.7	415/1207	34.4	0.253
Enacted	Physical violence*	1545/4804	32.2	518/1205	43.0	<0.001
Enacted	Forced to have sex*	1473/4799	30.7	459/1200	38.3	<0.001

*Not specified as attributable to sex work

Supplementary Table 3: Sex Work Related Sexual Behavior Stigma Measures

Stigma Measure (yes/no response)	Type of stigma
Have you ever felt excluded from family activities because you sell sex?	Perceived
Have you ever felt that family members have made discriminatory remarks or gossiped about you because you sell sex?	Perceived
Have you ever felt rejected by your friends because you sell sex?	Perceived
Have you ever felt afraid to go to health care services because you worry someone may learn you sell sex?	Perceived
Have you ever avoided going to health care services because you worry someone may learn you sell sex?	Anticipated
Have you ever felt that you were not treated well in a health center because someone knew that you sell sex?	Perceived
Have you ever heard health care providers gossiping about you (talking about you) because you sell sex?	Enacted
Have you ever been denied health services or had someone keep you from receiving health services because you sell sex?	Enacted
Have you ever felt that the police refused to protect you because you sell sex?	Perceived
Have you ever felt scared to be in public places because you sell sex?	Perceived
Have you ever been verbally harassed and felt it was because you sell sex?	Enacted
Have you ever been blackmailed by someone because you sell sex?	Enacted
Has someone ever physically hurt you (pushed, shoved, slapped, hit, kicked, choked or otherwise physically hurt you)?	Enacted
Have you ever been forced to have sex when you did not want to? (By forced, I mean physically forced, coerced to have sex, or penetrated with an object, when you did not want to).	Enacted

Supplementary Table 4: Sex work laws by country and legal categorizations

Country	Selling	Buying/soliciting	Organize	Legal Category	Citations
Burkina Faso	Not specified	Illegal	Illegal	Selling sex is not legally specified	(1)
Cameroon	Illegal	Illegal	Illegal	Fully Criminalized	(2-4)
Cote d’Ivoire	Illegal	Legal	Illegal	Partially legalized	(2, 5)
Guinea Bissau	Not specified	Not specified	Not specified	Selling sex is not legally specified	(6, 7)
Lesotho	Illegal	Illegal	Illegal	Fully criminalized	(8, 9)
Senegal	Legal*	Illegal	Illegal	Partially legalized	(2, 10)
South Africa	Illegal	Illegal	Illegal	Fully criminalized	(2)
Kingdom of eSwatini	Illegal	Illegal	Illegal	Fully criminalized	(11)
The Gambia	Illegal	Illegal	Illegal	Fully criminalized	(12)
Togo	Legal	Illegal	Illegal	Partially legalized	(13)

*Regulated through registration process

Supplementary references

1. Bureau of Democracy Human Rights and Labor. 2009 Country Reports on Human Rights Practices: Burkina Faso. US State Department 2010.
2. Sex Work Law: Sexuality Poverty and Law Programme; [cited 2018]. Available from: http://spl.ids.ac.uk/sexworklaw/countries.
3. Sexuality Poverty and Law Programme. S343 Cameroon Penal Act [cited 2018]. Available from: http://spl.ids.ac.uk/content/cameroon.
4. 2016/007. Sect. 343 and 294.
5. Country Reports on Human Rights Practices Cote d'Ivoire Bureau of Democracy Human Rights and Labor. US State Department 2009.
6. ChartsBin statistics collector team. The Legal Status of Prostitution by Country 2010 [cited 2019 30 April 2019]. Available from: http://chartsbin.com/view/snb.
7. Bureau of Democracy Human Rights and Labor. 2008 Country Reports on Human Rights Practices: Guinea Bissau. US State Department 2009.
8. Penal Code Act 2010, (2012).
9. UNDP. Report of the Assessment of the Legal Environment ofr HIV and AIDS in Lesotho2016.
10. Mgbako C, Smith LA. Sex Work and Human Rights in Africa. Fordham International Law Journal. 2011;33(4).
11. Criminal Law and Procedure ACT 6/1889.
12. Bureau of Democracy Human Rights and Labor. 2009 Country Reports on Human Rights Practices: The Gambia. US State Department 2010.
13. Bureau of Democracy Human Rights and Labor. 2009 Country Reports on Human Rights Practices: Togo. US State Department 2010.
