# Supplementary material for: The role of sex work laws and stigmas in increasing HIV risks among sex workers
Source: Nat Commun. 2020 Feb 18;11:773. doi: 10.1038/s41467-020-14593-6 (PMC7028952; doi:10.1038/s41467-020-14593-6)
Supplement: Supplementary file 3 — Reporting Summary [file 41467_2020_14593_MOESM3_ESM.pdf]

nature research

Corresponding author(s): DBPR NCOMMS-19-15147B

Last updated by author(s): 12/31/2019

Reporting Summary

Nature Research wishes to improve the reproducibility of the work that we publish. This form provides structure for consistency and transparency in reporting. For further information on Nature Research policies, see Authors & Referees and the Editorial Policy Checklist .

Statistics

For all statistical analyses, confirm that the following items are present in the figure legend, table legend, main text, or Methods section.

n/a	Confirmed	
☐	☒	The exact sample size (*n*) for each experimental group/condition, given as a discrete number and unit of measurement
☐	☒	A statement on whether measurements were taken from distinct samples or whether the same sample was measured repeatedly
☐	☒	The statistical test(s) used AND whether they are one- or two-sided<br>*Only common tests should be described solely by name; describe more complex techniques in the Methods section.*
☐	☒	A description of all covariates tested
☐	☒	A description of any assumptions or corrections, such as tests of normality and adjustment for multiple comparisons
☐	☒	A full description of the statistical parameters including central tendency (e.g. means) or other basic estimates (e.g. regression coefficient) AND variation (e.g. standard deviation) or associated estimates of uncertainty (e.g. confidence intervals)
☐	☒	For null hypothesis testing, the test statistic (e.g. *F*, *t*, *r*) with confidence intervals, effect sizes, degrees of freedom and *P* value noted<br>*Give P values as exact values whenever suitable.*
☒	☐	For Bayesian analysis, information on the choice of priors and Markov chain Monte Carlo settings
☒	☐	For hierarchical and complex designs, identification of the appropriate level for tests and full reporting of outcomes
☒	☐	Estimates of effect sizes (e.g. Cohen's *d*, Pearson's *r*), indicating how they were calculated

Our web collection on statistics for biologists contains articles on many of the points above.

Software and code

Policy information about availability of computer code

Data collection	All data were collected and stored using the data collection platform software SurveyCTO.
Data analysis	All analyses were conducted in Stata v.15.1. Custom code was used to pool the data and to analyze the data for this study.

For manuscripts utilizing custom algorithms or software that are central to the research but not yet described in published literature, software must be made available to editors/reviewers. We strongly encourage code deposition in a community repository (e.g. GitHub). See the Nature Research guidelines for submitting code & software for further information.

Data

Policy information about availability of data

All manuscripts must include a data availability statement. This statement should provide the following information, where applicable:

- Accession codes, unique identifiers, or web links for publicly available datasets
- A list of figures that have associated raw data
- A description of any restrictions on data availability

The de-identified data that support the findings of this study are available from the corresponding author upon reasonable request.

Field-specific reporting

Please select the one below that is the best fit for your research. If you are not sure, read the appropriate sections before making your selection.

☐ Life sciences ☒ Behavioural & social sciences ☐ Ecological, evolutionary & environmental sciences

For a reference copy of the document with all sections, see nature.com/documents/nr-reporting-summary-flat.pdf

Behavioural & social sciences study design

All studies must disclose on these points even when the disclosure is negative.

Study description	Primary data collection was conducted through ten country-specific studies led by one investigative team. Respondent driven sampling was used in each of the ten country-specific studies between 2011 and 2018. Data are quantitative and collected through interviewed administered socio-behavioral survey and biological testing for HIV. All country-specific studies are cross-sectional. Data were collected across 21 sites in 10 countries and were pooled for analysis.
Research sample	The research sample was female sex workers in Burkina Faso, Cameroon, Côte d'Ivoire, The Gambia, Guinea-Bissau, Lesotho, Senegal, eSwatini, South Africa, and Togo. Participants were eligible if they self-reported female sex assigned at birth; were 18 years or older; attributed more than half of their income in the past 12 months to selling sex; and were capable of providing informed consent. There was no maximum age restriction set for inclusion. Country-specific eligibility criteria included city or area of residence. All participants provided verbal or written informed consent. All country specific studies were reviewed approved by an ethical review board and related bodies in the country of data collection and the Johns Hopkins School of Public Health Institutional Review Board.
Sampling strategy	Respondent driven sampling was used to recruit individuals in each of the countries. Respondent driven sampling was used as a method to reach hidden, or hard-to-reach populations such as female sex workers. Recruitment chains were initiated through participants selected to be "seeds". Recruitment of seeds was based on convenience sampling, informed by formative research and a mapping exercise in each setting, with selection based on meeting the following criteria: understanding of recruitment process; able to recruit peers; knows many other peers; and shows interest in being a recruiter. Seeds were asked to recruit other female sex workers beginning a series of chain-referral sampling. Study staff explained the study to potential seeds and provided willing participants with contact information to arrange eligibility screening and interviews at the study site. After screening and providing informed consent, seeds completed an interviewer-administered socio-behavioral questionnaire and underwent biological testing for HIV. Thereafter, seeds were provided with three coupons to distribute to peers. Subsequent recruits were then provided with three coupons of their own to distribute within their peer network. This limited the number of people accrued by any one participant. With each additional wave, recruitment was expected to have become more representative and a closer approximation of a random sample. Subsequent seeds were released until the desired sample size was reached. Sample size calculations were determined for each country-specific data collection independently, based on expected response rate and power needed to assess indicators of interest.
Data collection	Potential participants arrived to a respective study site after being recruited through respondent driven sampling. All participants were assessed for eligibility and provided informed written or oral consent. Socio-behavioral questionnaires were administered by trained interviewers and conducted in the respective local language for each country, and measures collected through the interviews were self-reported by the participant. Interviews were conducted in a private room within the study site. Interviewers used tablets to collect the questionnaire responses and data. SurveyCTO was the data collection platform used to collect and store the data. Biological testing for HIV, including pre- and post-test counseling, was conducted by a trained professional and was administered consistently with country-specific national guidelines. No personal identifiers were collected. All data were stored on a secure, password protected server.
Timing	Data collection was conducted independently through country-specific studies between 2011 and 2018. All country-specific studies were cross sectional and data were collected across 21 sites in 10 countries. The time periods for data collection for each of the country-specific studies are: Burkina Faso (January–August 2013); Cameroon (November 2015 – October 2016); Côte d'Ivoire (March 2015–February 2016); The Gambia (May 2017 – May 2018); Guinea-Bissau (September – November 2017); Lesotho (February - September 2014); Senegal (February–November 2015); eSwatini (August - October 2011); South Africa (October 2014 – April 2015); and Togo (January–June 2013).
Data exclusions	No data were excluded from analysis. Participants were able to refuse to respond to questions in the survey, and therefore some indicators have missing data. Missing data were dropped from analysis.
Non-participation	Participants were recruited through respondent driven sampling, and therefore were recruited to participate through individuals within their social network. The number of individuals who declined to receive a recruitment coupon from another participant is not known. Additionally, coupons that were distributed but that did not result in a new participant were not captured to assess the number of participants who declined, or did not arrive to the study site.
Randomization	Participants were not allocated to experimental groups in this study. All data in this study were cross-sectional in nature, and no intervention was assessed.

Reporting for specific materials, systems and methods

We require information from authors about some types of materials, experimental systems and methods used in many studies. Here, indicate whether each material, system or method listed is relevant to your study. If you are not sure if a list item applies to your research, read the appropriate section before selecting a response.

Materials & experimental systems

n/a	Involved in the study
☒	☐ Antibodies
☒	☐ Eukaryotic cell lines
☒	☐ Palaeontology
☒	☐ Animals and other organisms
☐	☒ Human research participants
☒	☐ Clinical data

Methods

n/a	Involved in the study
☒	☐ ChIP-seq
☒	☐ Flow cytometry
☒	☐ MRI-based neuroimaging

Human research participants

Policy information about studies involving human research participants

Population characteristics

See above.

Recruitment

Participants were recruited through respondent driven sampling. Respondent driven sampling aims to utilize networks to reach marginalized or hard-to-reach populations. However, individuals who are less connected to a social network, and potentially more marginalized within that network may be underrepresented in this sample. This may underestimate stigma measures and poor health outcomes in our sample.

Ethics oversight

Country specific data collection were reviewed and approved by an ethical review board and related bodies in the country of data collection and the Johns Hopkins School of Public Health Institutional Review Board. Country specific ethic committees include: Health Research Ethics Committee of Burkina Faso, National Ethics Committee of Cameroon, the Health Research Ethics Committee of Côte d'Ivoire, National Research Ethics Committee of Guinea Bissau, the Lesotho National Health Research Ethics Committee, the Senegalese National Health Research Ethics, Institutional Review Boards of the Human Sciences Research Council in South Africa, the Swaziland Scientific Ethics Committee, Scientific Coordination Committee in the Gambia, the Ethical Committee of Togo.

Note that full information on the approval of the study protocol must also be provided in the manuscript.
